# Abdominal Aortic Endograft Implantation Immediately Induces Vascular Stiffness Gradients That May Promote Adverse Aortic Neck Dilatation: Results of A Porcine *Ex Vivo* Study

**DOI:** 10.1177/15266028231169178

**Published:** 2023-05-08

**Authors:** Isabel N. Schellinger, Jörg Naumann, Annett Hoffmann, Sarah-Jane Barnard, Sandra Düsing, Markus U. Wagenhäuser, Josephina Haunschild, Dierk Scheinert, Gerd Hasenfuß, Christian D. Etz, Uwe Raaz

**Affiliations:** 1 Department of Angiology, University Medical Center Leipzig, Leipzig University, Leipzig, Germany; 2 University Department for Cardiac surgery, Leipzig Heart Center, Leipzig, Germany; 3 Department for Endocrinology, Nephrology, Rheumatology, University Medical Center Leipzig, Leipzig University, Leipzig, Germany; 4 Department of General, Visceral, Transplant, Vascular and Pediatric Surgery, University Hospital Würzburg, Würzburg, Germany; 5 Department of Vascular and Endovascular Surgery, Medical Faculty and University Hospital Düsseldorf, Heinrich-Heine-University Düsseldorf, Düsseldorf, Germany; 6 Molecular and Translational Vascular Medicine, Department of Cardiology and Pneumology, Heart Center at the University Medical Center Göttingen, Göttingen, Germany; 7 Partner site Göttingen, German Center for Cardiovascular Research e.V., Göttingen, Germany

**Keywords:** abdominal aortic aneurysm, EVAR, endograft, aortic neck dilatation, aortic stiffness, aortic remodeling

## Abstract

**Purpose:**

Endovascular aortic repair (EVAR) is the method of choice for most abdominal aortic aneurysm (AAA) patients requiring intervention. However, chronic aortic neck dilatation (AND) following EVAR progressively weakens the structural seal between vessel and endograft and compromises long-term results of the therapy. This experimental *ex vivo* study seeks to investigate mechanisms of AND.

**Materials and Methods:**

Porcine abdominal aortas (n=20) were harvested from slaughterhouse pigs and connected to a mock circulation. A commercially available endograft was implanted (n=10) or aortas were left untreated as controls (n=10). Vascular circumferential strain was assessed via ultrasound in defined aortic segments as a parameter of aortic stiffness. Histology and aortic gene expression analysis were performed to investigate potential changes of aortic wall structure and molecular differences due to endograft implantation.

**Results:**

We found that endograft implantation acutely induces a significant stiffness gradient directly at the interface between stented and unstented aortic segments under pulsatile pressure. Comparing stented aortas with unstented controls, we detected increased aortic expression levels of inflammatory cytokines (*Il6* and *Ccl2*) and matrix metalloproteinases (*Mmp2* and *Mmp9*) after 6 hours of pulsatile pressurization. This effect, however, was abolished when repeating the same experiment under 6 hours of static pressure.

**Conclusions:**

We identified endograft-induced aortic stiffness gradients as an early trigger of inflammatory aortic remodeling processes that might promote AND. These results highlight the importance of adequate endograft designs to minimize vascular stiffness gradients and forestall late complications, such as AND.

**Clinical Impact:**

AND may compromise the long-term results following endovascular aortic repair. However, the mechanisms behind the underlying detrimental aortic remodeling are still unclear. In this study we find that endograft-induced aortic stiffness gradients induce an inflammatory aortic remodeling response consistent with AND. This novel pathomechanistic insight may guide the design of new aortic endografts that minimize vascular stiffness gradients and forestall late complications such as AND.

## Introduction

Abdominal aortic aneurysm (AAA) is a disease of the aging aorta mainly affecting male patients above the age 60 years.^1^ The chronic progressive nature of the disease is characterized by a steadily increasing, size-dependent risk of fatal AAA rupture. Thus, current guidelines recommend elective AAA repair in aneurysms exhibiting a maximum diameter of 5 to 5.5 cm.^2,3^

Today, endovascular aortic repair (EVAR) is used as the method of choice in up to 80% of all elective AAA patients.^3–5^ This is mainly due to a less invasive character of EVAR compared with traditional open aortic repair (OR) that translates into faster hospital discharge and lower 30-day morbidity and mortality.^6–8^ However, recent studies indicate that the early survival benefits of EVAR are lost after only 1 to 5 years postintervention, and moreover, are reversed in the long term with increased mortality compared with OR after 8 years.^9–12^

Secondary aneurysm growth and sac rupture is a main factor driving late mortality in EVAR patients.^12^ Mechanistically, gradual aortic neck dilatation (AND)—that is found in up to 25% of EVAR patients during follow-up—may cause a loss of seal and insufficient aneurysm exclusion with subsequent endoleak development and/or graft migration.^13^ The cause of AND, however, still is unclear and may potentially be due to the mechanical interaction between endograft and aorta or, alternatively, be part of the underlying aneurysmal disease. In the former case, adequate endograft sizing and design may help to prevent AND as well as resultant EVAR complications.

Here, using an *ex vivo* porcine aortic model, we provide first mechanistic evidence that aortic endograft implantation induces a mechanical stiffness gradient between adjacent stented and unstented vessel segments resulting in an early aortic gene response that may drive AND.

## Materials and Methods

### Preparation of Aortic Specimens

Abdominal aortas were harvested at a local slaughterhouse from healthy hybrid pigs slaughtered for commercial food production (Duroc, German landrace, Piétrain; 7–8 months; 150–170 kg). Aortas were transported in ice-cold histidine-tryptophan-ketoglutarate (HTK) solution and used for experiments within a few hours. Before experimental use of aortic segments, all side branches were ligated. Dissection of aortic (peri)adventitial tissue was kept to a minimum to preserve *in vivo* conditions as much as possible.

### Aortic Mechanical Stimulation *ex vivo*

**Figure 1.**
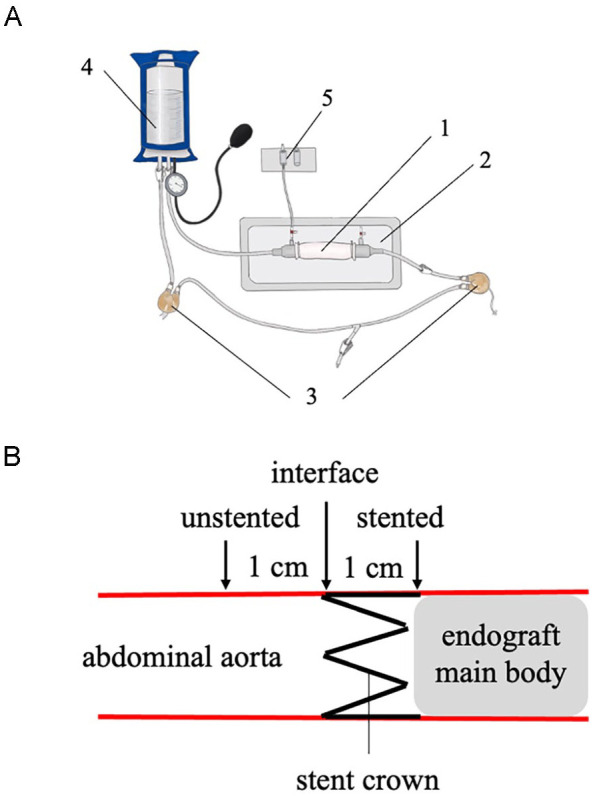
Experimental setup. (A) Aortic mechanical stimulation. Aortic segments (1) were placed in a vessel chamber (2) and connected to a mock circulation was driven by commercially available pump units (EXCOR 60 mL, Berlin Heart GmbH, Germany) (3). Pressure levels were adjusted via pneumatic “pressure bag” (VBM Medizintechnik, Germany) (4) serving as system afterload and continuously monitored via pressure transducers (5). (B) Axial location of ultrasound measure points. Strain quantifications via ultrasound M-Mode were performed at the stented, unstented, and interface segments of the aortic vessel.

For *ex vivo* mechanical stimulation, the abdominal aortas were cannulated and connected to a mock circulation system as depicted in Figure 1A. Aortas were mounted in a heated vessel chamber and physiological saline solution (PSS) at 37°C, aerated with 5% CO_2_/95% O_2_ was used to fill the vessel chamber and for aortic perfusion. To compensate for the elastic axial recoil of the aortas after harvesting, aortas were then briefly subjected to a static pressure of 200 mm Hg and axial pre-stretch was increasingly applied until no vessel buckling was observed. For aortic sizing, a static pressure of 100 mm Hg (representing the mean aortic pressure *in vivo*) was applied, and diameters of the aortic segment were quantified at 3 distinct locations: proximally in the suprarenal segment (where later the stent crown of the endograft was placed), as well as 2 cm (medial) and 4 cm (distal) below this point. Following this maneuver, implantation of the aortic endograft (Zenith Alpha^™^ Abdominal Endovascular Graft, Main Body, 22-mm graft diameter, 70-mm graft length; Cook Medical) was performed in the respective experimental groups. To preserve the biomechanical characteristics of the endograft we refrained from multiple explantation, re-crimping, and re-implantation maneuvers, and used the same endograft for 1 experiment only. The proximal part of the stent-graft (stent crown) was implanted proximally of the level of the renal arteries with only little variations between animals. Subsequently, aortas were exposed to either a static intravascular pressure of 130 mm Hg for 6 hours or a pulsatile intravascular pressure of 130/80 mm Hg (heart rate=80/min) for 6 hours, respectively. Thus, the study comprised 4 experimental groups:

1. Aortas with endograft subjected to pulsatile pressure.
2. Aortas without endograft subjected to pulsatile pressure (pulsatile pressure controls).
3. Aortas with endograft subjected to static pressure.
4. Aortas without endograft subjected to static pressure (static pressure controls).

Static and pulsatile pressure conditions were applied to differentiate static effects of the endograft implantation (e.g., effects related to the endograft implantation itself, static impact of radial force) from effects of altered pulsatile aortic wall motion (e.g., impact of altered wall stiffness due to endograft insertion). After conclusion of the experiment, aortas were removed from the cannulas and processed for histology or RNA isolation, respectively.

### Aortic Imaging

Aortic *ex vivo* imaging was performed by 1 dedicated sonographer using GE Vivid i ultrasound system (GE Medical, USA) with a linear transducer (8L-RS). The abdominal aorta was visualized in B-mode in a longitudinal orientation. M-mode images tracking the anterior and posterior aortic wall motion were then recorded in a perpendicular orientation at 3 predefined axial locations (stented segment, interface segment, and unstented segment; Figure 1B). The interface segment denotes the immediate transition of the stented and unstented vessel; the stented segment is located 1 cm distally in the stented aorta and the unstented segment is located 1 cm proximally in the unstented aorta (Figure 1B). Multiple cardiac cycles (at least 4) were recorded and the acquired images were stored digitally. Image analysis was performed using the integrated software package of the ultrasound system. Systolic diameter (Ds) and diastolic diameter (Dd) were quantified, and circumferential cyclic strain ε was calculated as a metric of aortic stiffness: ε = (Ds–Dd)/Dd×100%. Stiffness (strain) ratio (SR) between segments was calculated as SR = ε _segment 1_ / ε _segment 2_.

### Tissue Preparation

After conclusion of the *ex vivo* experiments aortic segments of interest were immediately cut into half. Subsequently, one randomly chosen half was processed for histology (n=5/experimental condition) and the other half for gene expression analysis (n=5/experimental condition).

### Tissue Preparation for Histologic Analysis

After conclusion of the *ex vivo* experiments, aortic specimens were immediately fixed in 10% formalin, frozen at 4°C in a refrigerator, and stored for 24 hours. Then, the tissue samples were processed into paraffin-embedded blocks.

### Stainings

Aortic cross-sections (7 μm) were cut from each paraffin block for histological staining. The sections were stained with the hematoxylin and eosin (HE) according to standard protocols to evaluation the overall structure of the aortic well. Additional staining with Elastica von Gieson (EvG) was performed for visualization of aortic elastic fibers.

### Tissue Preparation for Gene Expression Analysis

After conclusion of the *ex vivo* experiments, aortic specimens were snap-frozen in liquid nitrogen and stored at –80°C before further processing.

### RNA Isolation and Quantification

Total RNA was isolated using a TRIzol-based (Invitrogen) RNA isolation protocol. RNA was quantified by Nanodrop (Agilent Technologies), and RNA quality was verified using an Agilent 2100 Bioanalyzer (Agilent Technologies). Samples required 260/280 ratios >1.8, and sample RNA integrity numbers >9 for inclusion. The iScript cDNA synthesis kit (Bio-Rad) was used to synthesize first-strand cDNA according to the manufacturer’s protocol. TaqMan quantitative reverse transcription polymerase chain reaction (qRT-PCR) assay was performed using porcine-specific primers for *Il6, Ccl2, Mmp2*, and *Mmp9* (Life Technologies). All probes were normalized to 18S as internal control. Amplification took place on a QuantStudio12K Flex (Applied Biosystems). All fold changes were calculated by the method of ΔΔCt.

### Statistics

Data are presented as mean±SEM. For comparison of 2 groups, Student’s t-test (2-tailed) was performed; multiple groups (≥3 groups) comparison was accomplished by analysis of variance (ANOVA) with Bonferroni’s post-test. Normality and homoscedasticity were tested to ensure that parametric testing was appropriate. A value of p<0.05 (2-sided) was considered statistically significant.

## Results

### Native Porcine Abdominal Aortas Exhibit A Homogeneous Stiffness Profile

**Figure 2.**
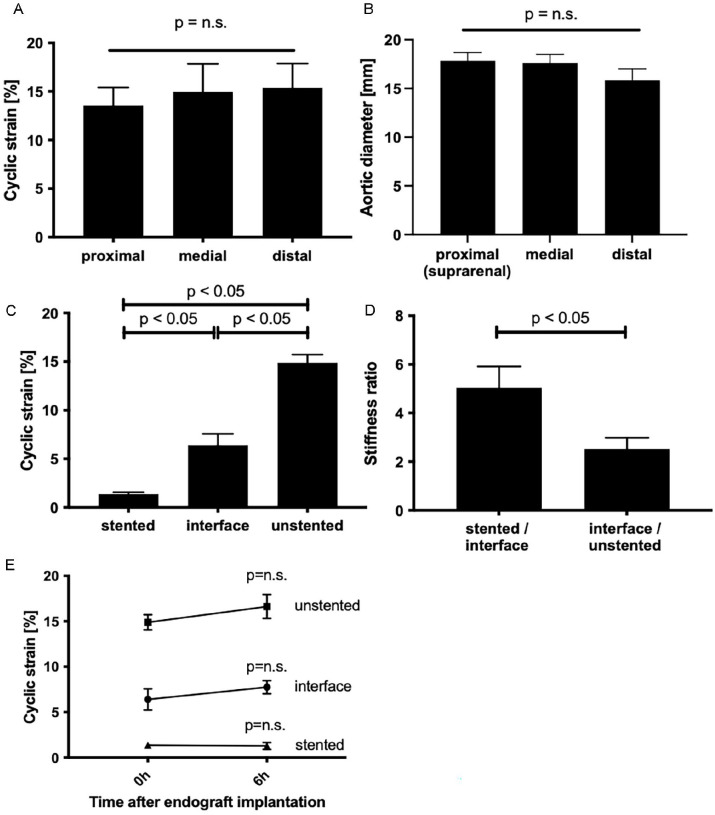
Aortic endograft implantation immediately induces a permanent axial stiffness gradient at the interface between stented and unstented aorta. (A) Cyclic circumferential strain measured in 2-cm interspaced native porcine abdominal aortic segments under 130/80 mm Hg pulsatile pressurization. The proximal measure point refers to the aortic segment proximally of the renal arteries. This also corresponds to the site where later the stent crown of the endograft was implanted. The medial and distal measure points were arbitrarily chosen 2 cm distally and 4 cm distally of the proximal point, respectively (n=5). Values are mean±Standard Error of the Mean (SEM). (B) Aortic diameters measured in 2-cm interspaced native (unstented) porcine abdominal aortic segments under 100 mm Hg static pressure. The proximal measure point refers to the aortic segment proximally of the renal arteries. This also corresponds to the site where later the stent crown of the endograft was implanted. The medial and distal measure points were arbitrarily chosen 2 cm distally and 4 cm distally of the proximal point, respectively (n=5). (C) Cyclic circumferential strain measured after endograft implantation in stented, interface, and unstented aortic segments under pulsatile pressurization (n=5). Values are mean±SEM. (D) Stiffness gradient (expressed as stiffness ratio) between stented and interface aortic segments vs interface and unstented segments following aortic endograft implantation (n=5). Values are mean±SEM. (E) Time course of cyclic circumferential strain in unstented, stented, and interface aortic segments immediately after endograft implantation (0 hour) and after 6 hours of pulsatile pressurization (6 hours) (n=5). Values are mean±SEM. p=ns indicates non-significance vs 0 hour.

In first set of experiments, we quantified baseline vascular stiffness of native (unstented) abdominal aortic segments under pulsatile (130/80 mm Hg) pressure. We found that aortic cyclic circumferential strain (used as a measure of aortic stiffness) was homogenous along the axial length of the abdominal aortic segment studied (n=5 individual aortas; Figure 2A).

### Endograft Implantation Immediately Induces an Axial Stiffness Gradient at the Interface Between Stented and Unstented Aortas

In a next step, the stent-graft was introduced in the aorta. Implantation of the endograft’s stent crown (nominal diameter: 22 mm) at the proximal (suprarenal) aortic fixation point (aortic native diameter: 18 mm; Figure 2B) corresponded to an oversizing of ~22%, that was consistent with the device’s instructions for use (IFU). Under pulsatile pressurization (130/80 mm Hg), aortic strain was then measured in the stented and unstented segments as well as at the immediate interface between both zones (Figure 1). Cyclic aortic strain quantification revealed aortic stiffness was markedly reduced in the stented segment compared with both the interface segment and unstented aorta (n=5 individual stented aortas; Figure 2C). Furthermore, the interface segment was significantly stiffer than the distal unstented segment (Figure 2C). Of note, the largest stiffness gradient (SR) was found between stented and interface segment (n=5 individual stented aortas; Figure 2D). In addition, observing cyclic strain over time, aortic stiffness and gradients did not change significantly in the respective segments over 6 hours (n=5 individual stented aortas; Figure 2E).

### Endograft-Induced Stiffness Gradients Upregulate Pro-Inflammatory Genes and Extracellular Matrix Genes in the Aortic Wall

**Figure 3.**
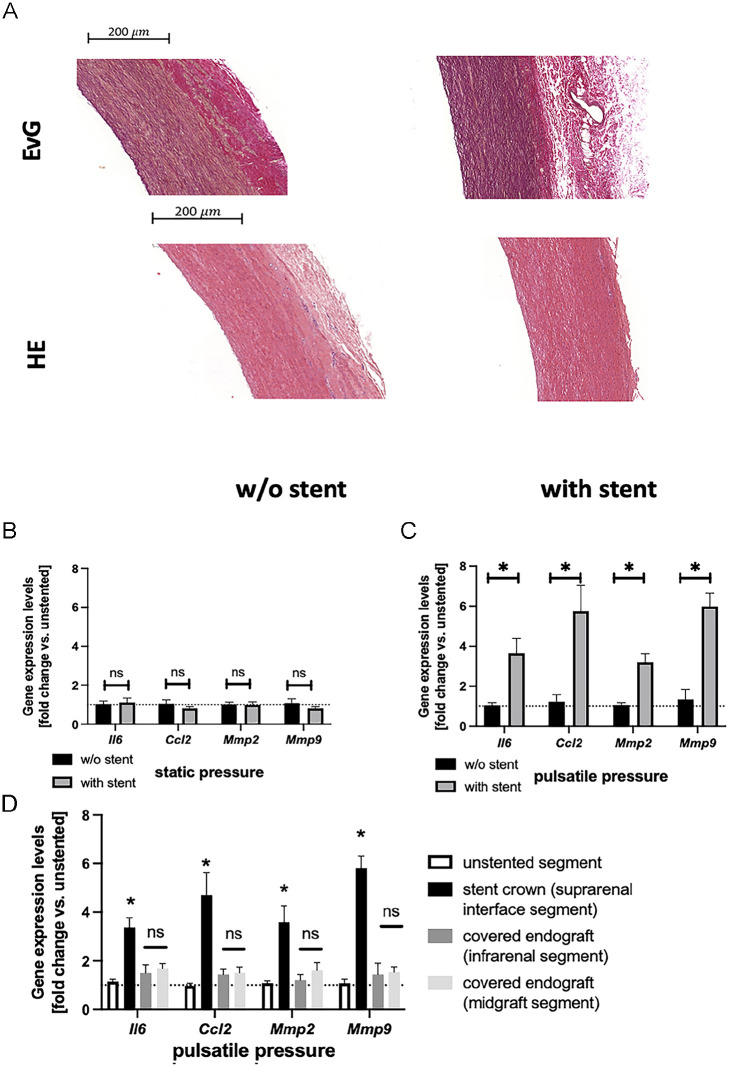
Endograft-induced stiffness gradient induces inflammatory and extracellular matrix-modulating genes in the aortic wall. (A) Representative images of aortic cross-sections of native vs stented aortas (interface segment) stained with hematoxylin and eosin (HE) and Elastica von Gieson (EvG; elastic lamellae are stained dark purple). Aortas were exposed to 6 hours of pulsatile pressurization. (B) Expression levels of *Il6, Ccl2, Mmp2*, and *Mmp9* in the interface segment of stented aortas vs unstented aortas following 6 hours of static pressurization (n=5/group). Values are mean±SEM and expressed as fold changes relative to the mean expression level in unstented aortas (=1; dotted line). ns indicates non-significance. (C) Expression levels of *Il6, Ccl2, Mmp2*, and *Mmp9* in the interface segment of stented aortas vs unstented aortas following 6 hours of pulsatile pressurization (n=5/group). Values are mean±SEM and expressed as fold changes relative to the mean expression level in unstented aortas (=1; dotted line). * indicates p<0.05 vs unstented. (D) Aortic expression levels of *Il6, Ccl2, Mmp2*, and *Mmp9* at distinct axial locations along the endograft (as indicated) following 6 hours of pulsatile pressurization (n=5/group). Values are mean±SEM and expressed as fold changes relative to the mean expression level of a native (unstented) aortic segment (=1; dotted line). * indicates p<0.05 vs unstented segment, ns indicates non-significant vs unstented segment.

We next sought to investigate the potential role of endograft-induced stiffness gradients to trigger aortic remodeling processes resulting in AND. Thus, we first compared wall structure of aortas with vs without endograft implantation following 6 hours of pulsatile pressurization. Here, both HE and EvG histologic staining revealed no significant differences between treatment conditions (Figure 3A).

In the absence of gross histologic differences, we next analyzed aortic gene expression as subtle markers indicative of an early inflammatory response (*Il6* and *Ccl2*) and aortic wall remodeling (*Mmp2* and *Mmp9*). We first compared expression profiles of stented vs unstented vessels after 6 hours of static (130 mm Hg) pressurization. Here, we found no difference between stented (n=5 individual aortas) and unstented (n=5 individual aortas) conditions (Figure 3B). However, following 6 hours of pulsatile pressurization, gene expression was markedly upregulated in the interface zone of stented aortas (n=5) compared with identically treated unstented vessels (n=5)—indicating a functional significance of the endograft-induced stiffness gradient (Figure 3C). Elevated gene expression levels were confined to the stent crown (interface segment) and did not extend distally to the covered endograft segments (Figure 3D).

## Discussion

Aortic neck dilatation is a phenomenon frequently observed in AAA patients following EVAR graft implantation that jeopardizes sufficient AAA exclusion and worsens clinical outcomes. The pathophysiology behind AND is still unclear and may be a further manifestation of the underlying AAA disease *per se* or, alternatively, be due to the endograft implantation.

This study provides first mechanistic evidence that vascular stiffness gradients following aortic endograft implantation may trigger molecular inflammatory aortic remodeling processes potentially promoting AND.

To investigate the mechanical interaction between endograft and aorta and consecutive molecular events, we employed an *ex vivo* experimental setup exposing freshly harvested porcine abdominal aortas to controlled hemodynamic conditions. Here, physiologic pulsatile pressure (130/80 mm Hg) induced an aortic circumferential strain of ~13% (Figure 2A), that is, consistent with human *in vivo* values reported in other studies.^14–16^

Endograft implantation immediately and permanently increased aortic stiffness (i.e., reduced cyclic aortic strain) in the stented aortic segment (Figure 2B). This observation is in line with previous *ex vivo* studies and with clinical data showing increased arterial stiffness in patients having undergone thoracic endovascular aneurysm repair (TEVAR) or EVAR procedures.^17,18^ Importantly, we also found a significant stiffness gradient directly at the interface between stented and unstented aorta—a region that anatomically corresponds to the aortic neck in AAA patients (Figure 2C). Of note, our experiments were performed with an adequately sized endograft (oversizing of ~22%). It is conceivable that excessive oversizing of the endograft may further stretch the vessel wall leading to enhanced mechanical stiffening and further increased stiffness gradients. However, in previous studies, different stent-graft oversizing schemes did not significantly influence the amount of aortic strain reduction.^17^ Still, inadequate sizing of the endograft should be avoided to limit the mechanical stress imposed on the aorta.

We previously reported the mechanistic significance of aortic stiffness gradients to drive inflammatory aortic remodeling.^14^ Specifically, we found that inhomogeneous aortic wall motions (due to stiffness mismatches) induce adverse mechanical forces that promote aneurysm formation.^14^ Thus, we were intrigued by the hypothesis that the observed endograft-induced aortic stiffness grants my trigger aortic remodeling eventually resulting in AND.

To further investigate this hypothesis, we first analyzed the aortic wall of endograft-treated vessels at the conclusion of the *ex vivo* protocol. Histology, however, did not reveal signs of aortic remodeling in comparison with untreated aortas. This was somewhat expected as 6 hours of total experimental time may be too short to bring about structural aortic changes, particularly in an *ex vivo* setting.

Therefore, in the absence of structural alterations, we looked for an early functional response indicative of aortic remodeling processes following endograft implantation. In this context, we previously found that gene expression of matrix metalloproteinases, *MMP-2* and *MMP-9*, as well as proinflammatory cytokines interleukin (*IL*)-*6* and *CCL2*—all of which play a role in aneurysmal wall remodeling—is rapidly induced in the aortic wall under adverse mechanical stress.^14^ Matrix metalloproteinases, MMP-2 and MMP-9, are produced by aortic smooth muscle cells (SMCs) and mediate the breakdown of aortic matrix macromolecules (such as fibrillar collagen and elastin). In addition, cytokines IL-6 and CCL2 released by SMCs may both promote the inflammatory milieu that characterizes aneurysmal disease.^1^ Indeed, following 6 hours of pulsatile pressurization expression levels of *Mmp2, Mmp9, Il6*, and *Ccl2* were all significantly upregulated in stented aortas compared with unstented controls (Figure 3C), and this effect was spatially confined to the stiffness transition zone (interface zone) at the proximal end of the endograft (stent crown) (Figure 3D). We next investigated whether gene expression was induced due to the mere presence of an endograft or rather due to the device-induced aortic stiffness gradient. Comparing expression levels between stented and unstented vessels after 6 hours of static pressurization (when stiffness gradients functionally do not come into effect) revealed no significant differences between treatments (Figure 3B). These data indicate that endograft-induced mechanical stiffness gradients—not the endograft implantation *per se*—may promote adverse aortic gene expression contributing to AND.

This study comes with several implications. There is multiple evidence that aortic endograft implantation immediately impairs the aortic *Windkessel* function resulting in adverse systemic hemodynamic effects.^19^ As such, previous studies found (T)EVAR procedures to increase aortic stiffness thereby elevating cardiac afterload resulting in left-ventricular hypertrophy and diastolic dysfunction.^20^ In addition, our data suggest that aortic stiffening following EVAR may also locally promote aortic pathologies, such as AND, independently of an underlying dilating aneurysmal disease. In this context, AND has usually been attributed to the endograft’s static radial force acting on the vessel wall. In contrast, this study provides new evidence that dilatative aortic remodeling post EVAR may rather be induced by an aortic stiffness gradient occurring at the interface between stented and unstented aorta. This should be considered in the design and development of new generations of aortic endografts to minimize stiffness gradients and should take into account factors, such as stent configurations, materials and fixation mechanisms.^21^ In this regard, while generally increasing aortic stiffness, polytetrafluoroethylene (PTFE)-covered stent-grafts were found to increase aortic pulse-wave velocity (as a marker of aortic stiffness) less than polyester covered stent-grafts that also was associated with some clinical benefit.^19,22^ Interestingly, another study found specific endograft designs to differentially influence aortic neck stiffness suggesting that the use of proximal “attachment barbs” for improved aortic fixation may restrain aortic wall motion and thereby increase vessel stiffness.^23^ Generally, this may indicate an engineering dilemma when maximal endograft fixation and durability needs to be counterbalanced against minimal interference with aortic mechanics (compliance).

In addition, we found that the endograft-induced stiffness gradient produces an aortic inflammatory gene response. This may locally contribute to a systemic inflammatory “post-implantation syndrome” (PIS) that is frequently observed after EVAR^24^ and may partly explain why stiffer polyester-based endografts carry a higher risk for PIS than PTFE-covered endografts.^19^

The main limitations of this study are inherent in its acute *ex vivo* design that may restrict an extrapolation of the experimental data into the clinical context. While our mock circulatory system enables a controlled dissection of hemodynamic effects on the vasculature—that are impossible in *in vivo* studies—it ignores many factors that may very well contribute to the clinical phenomenon (AND) under investigation (e.g, use of PSS instead of blood) and cannot fully recapitulate complex biological processes such as vascular remodeling that develop in intact organisms over longer periods of time. In fact, given the limited experimental duration of 6 hours, we deliberately chose aortic gene expression as an endpoint of the study, as a sustained gene expression response due to a mechanically altered milieu typically develops within a few hours.^14^ On the upside, however, due to the control of experimental variables, such as hemodynamics, *ex vivo* experiments provide a unique opportunity to investigate questions that cannot not be addressed *in vivo*. In fact, the differential investigation of the endograft under both static and pulsatile hemodynamics allowed us to separate effects arising out of the endograft’s stiffening of the vessel wall from effects due to the implantation itself. Other limitations of this study are the use of only 1 specific make/type of aortic endograft (Zenith Alpha, Cook Medical; a polyester based endograft that uses a stent crown with comparatively high radial forces for suprarenal fixation)^25^ that may restrict a generalization. However, this study may very well provide a mechanistic basis for further *in vivo* studies.

## Conclusion

In conclusion, this study provides first mechanistic evidence that an endograft-induced stiffness gradients at the interface between stented and unstented aorta produces a proinflammatory milieu potentially favoring adverse aortic remodeling processes under pulsatile hemodynamic load. This finding may further underline the need for a prioritized development of compliant endografts to better respect aortic mechanics/physiology.
